# A Case of Type 1 Congenital Pulmonary Airway Malformation (CPAM) in an Eight-Year-Old Girl Without a History of Recurrent Pneumonia or Respiratory Failure, Presenting With a Pulmonary Abscess

**DOI:** 10.7759/cureus.93885

**Published:** 2025-10-05

**Authors:** Ryutaro Ohira, Natsuho Adachi, Shoichiro Kanda, Amane Yamamoto, Motoki Ebihara, Tomo Kakihara, Jun Fujishiro, Keiichi Takizawa

**Affiliations:** 1 Pediatrics, Graduate School of Medicine, The University of Tokyo, Tokyo, JPN; 2 Pathology, Graduate School of Medicine, The University of Tokyo, Tokyo, JPN; 3 Pediatric Surgery, Graduate School of Medicine, The University of Tokyo, Tokyo, JPN

**Keywords:** congenital pulmonary airway malformation (cpam), pediatric pulmonology, pediatric surgery, pulmonary abscess, type 1 cpam

## Abstract

Congenital pulmonary airway malformation (CPAM) is a rare developmental anomaly of the lung, most often diagnosed in infancy or early childhood due to recurrent pneumonia or respiratory distress. We report the case of an eight-year-old girl who presented with fever, cough, hemoptysis, and chest pain, and was ultimately found to have pulmonary abscesses in the right upper and middle lobes. She had no prior history of recurrent respiratory infections or neonatal distress. Chest computed tomography revealed multilocular cystic lesions involving two lobes, raising suspicion for CPAM. The patient received prolonged intravenous and oral antibiotic therapy, followed by surgical resection consisting of a right upper lobectomy and wedge resection of the middle lobe. Histopathological examination confirmed Type 1 CPAM. She has remained free of recurrence since surgery. This case is notable for the unusually late presentation of Type 1 CPAM in school-aged children without prior symptoms, discovered only after the development of pulmonary abscesses. The absence of recurrent pneumonia or abnormal imaging made differentiation from a primary pulmonary abscess challenging. Given the risks of recurrent infections and malignant transformation, including mucinous adenocarcinoma and pleuropulmonary blastoma, surgical resection remains the preferred treatment, even in atypical or incidentally detected cases. This report underscores the importance of considering congenital cystic lung disease in the differential diagnosis of pediatric pulmonary abscess and highlights the role of thorough evaluation and timely surgical management in achieving favorable outcomes.

## Introduction

Congenital pulmonary airway malformation (CPAM), one of the congenital cystic lung diseases (CCLD), occurs in approximately 1 in 25,000-35,000 live births [1]. CPAM is a hamartomatous lesion resulting from abnormal airway development during lung morphogenesis and is classified into five subtypes (Types 0-4) based on the presumed site of origin within the respiratory tract [2]. Among these, Type 1, which arises at the bronchial level, is the most common, accounting for about 60%-70% of cases [1]. Type 1 CPAM is characterized by relatively large cysts (2-10 cm in diameter), which may cause mediastinal shift during the fetal period and respiratory distress after birth [2]. Even when not detected at birth, Type 1 CPAM is often diagnosed in infancy or early childhood due to complications such as pneumonia or lung abscess [3].

Here, we report a rare case of an eight-year-old girl diagnosed with a secondary pulmonary abscess associated with Type 1 CPAM, despite no prior history of cyst infection. This case is notable for the incidental discovery of a large multilobar cystic lesion during school age, in the absence of preceding symptoms. Recognition of CPAM as the underlying condition enabled a comprehensive treatment strategy, including surgical intervention after resolution of the abscess. We describe the diagnostic process and therapeutic approach in this unusual presentation.

## Case presentation

The patient was an eight-year-old girl with no significant past medical history, including no previous episodes of pneumonia or hospitalizations. During the perinatal period, oligohydramnios had been noted; however, no fetal abnormalities were reported. Her family history was notable for breast cancer in her maternal grandmother and malignant lymphoma in her maternal grandfather.

Two months before admission to our hospital, the patient developed fever, cough, and nasal discharge. She visited a local clinic and experienced temporary improvement with antibiotic treatment; however, her symptoms recurred intermittently. Nine days before admission, she developed a fever exceeding 39 °C, accompanied by nasal discharge, abdominal pain, and vomiting. Six days before admission, she revisited the clinic and was prescribed azithromycin. Five days before admission, she began to complain of midline chest pain, and four days before admission, her nocturnal cough worsened. Three days before admission, she returned to the clinic, where a rapid *Mycoplasma *antigen test was negative. Blood tests revealed a markedly elevated inflammatory response, with a C-reactive protein (CRP) level of 13.43 mg/dL. The patient exhibited decreased activity and mild dehydration. A chest X-ray showed extensive pneumonia in the right lung, and her CRP level had further increased to 24.2 mg/dL. Based on these findings, she was diagnosed with bacterial pneumonia and admitted to the referring hospital, where she was started on ampicillin-sulbactam (ABPC/SBT) at a dose of 150 mg/kg/day. The day before transfer to our hospital, a chest computed tomography (CT) scan revealed multiple abscesses in the right upper lobe. Given the need for further evaluation, the patient was transferred to our institution.

On physical examination at admission, the patient’s height was 120.0 cm and weight was 22.0 kg. Her vital signs were as follows: body temperature, 36.2 °C; heart rate, 122 beats per minute; blood pressure, 101/78 mmHg; and oxygen saturation (SpO₂), 96% on room air. She appeared slightly lethargic. Breath sounds were clear, though mildly diminished in the right upper lung field. Cardiac auscultation revealed a regular rhythm without murmurs. Laboratory tests showed elevated white blood cell and platelet counts, with a CRP level of 6.66 mg/dL, indicating persistent inflammation (Table 1).

**Table 1:** Laboratory and immunological findings on admission Con-A: concanavalin A, PHA: phytohemagglutinin, DHR: dihydrorhodamine.

Parameters	Value	Reference range
Complete blood count		
White blood cell count (×10³/µL)	19.8	4.5-13.5
Hemoglobin (g/dL)	13.6	11.5-15.5
Hematocrit (%)	41.9	35-45
Platelet count (×10⁴/µL)	64.3	15-45
Serum chemistry		
Total protein (g/dL)	7.8	6.0-8.0
Albumin (g/dL)	3.3	3.5-5.0
Aspartate aminotransferase (U/L)	31	10-40
Alanine aminotransferase (U/L)	20	10-35
Lactate dehydrogenase (U/L)	326	120-300
Alkaline phosphatase (U/L)	165	150-420
Blood urea nitrogen (mg/dL)	3.1	7.0-20
Creatinine (mg/dL)	0.29	0.29-0.53
Sodium (mmol/L)	138	135-145
Potassium (mmol/L)	4.7	3.5-5.0
Chloride (mmol/L)	100	98-107
C-reactive protein (mg/dL)	6.66	<0.3
Immunoglobulin G (mg/dL)	1548	700-1600
Immunoglobulin A (mg/dL)	466	40-400
Immunoglobulin M (mg/dL)	137	40-230
(1→3)-β-D-glucan (pg/mL)	9.1	<20
Tuberculosis test		
T-SPOT test	Negative	Negative
Lymphocyte function test		
Con-A stimulation index (cpm)	22,700	20,300-65,700
PHA stimulation index (cpm)	26,700	20,500-56,800
Lymphocyte subset analysis by flow cytometry		
CD3+ T cells (% of lymphocytes)	78.2	60-80
CD19+ B cells (% of lymphocytes)	19.4	10-20
DHR-123 flow cytometric assay (oxidative burst test)		
DHR-123 (unstimulated, PMA–) (%)	1.33	<5
DHR-123 (stimulated, PMA+) (%)	99.52	>95

**Figure 1:**
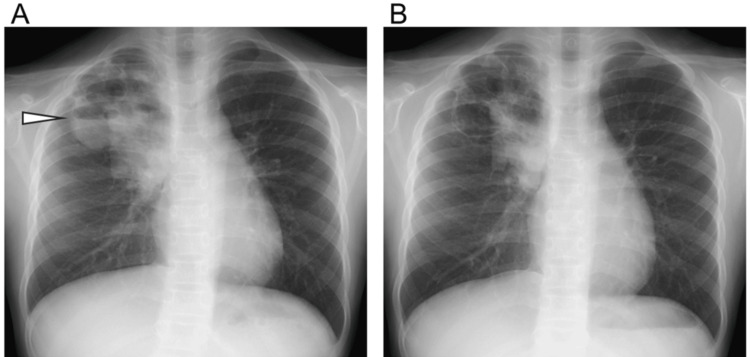
Chest X-ray images (A) On hospital day 11, infiltrative shadows and cavitary lesions were observed, with a fluid level visible within the cavitary lesion (arrowhead). (B) On hospital day 19, the infiltrative shadows had decreased in size but remained present, and the fluid level within the cavitary lesion had disappeared.

At the time of transfer, the patient was afebrile and her appetite had recovered; however, she continued to experience coughing and hemoptysis. Given the presence of pulmonary abscesses, intravenous ABPC/SBT was administered at a dose of 200 mg/kg/day for a total duration of four weeks. On hospital day 11, chest X-ray findings showed that the infiltrative and cavitary lesions had decreased in size compared with the previous imaging from the referring hospital. However, a fluid level had developed within the cavitary lesion. By hospital day 19, residual infiltrative shadows, likely representing part of the abscess, remained on the chest X-ray, but the fluid level within the cavity had disappeared (Figure 1).

To investigate the underlying cause of the pulmonary abscess, infection, immunodeficiency, and CCLD were considered as potential etiologies, and a thorough evaluation was conducted. Regarding the causative pathogen, blood and sputum cultures had not been obtained before antibiotic administration at the referring hospital. However, a nasal swab culture identified *Haemophilus influenzae*. There was no elevation in β-D-glucan, and the T-SPOT test was negative, making fungal infection and tuberculosis unlikely. Furthermore, a comprehensive immunological assessment revealed no evidence of antibody production defects, T- or B-cell deficiency or dysfunction, or neutrophil functional impairment, effectively excluding primary immunodeficiency disorders (Table 1).

**Figure 2:**
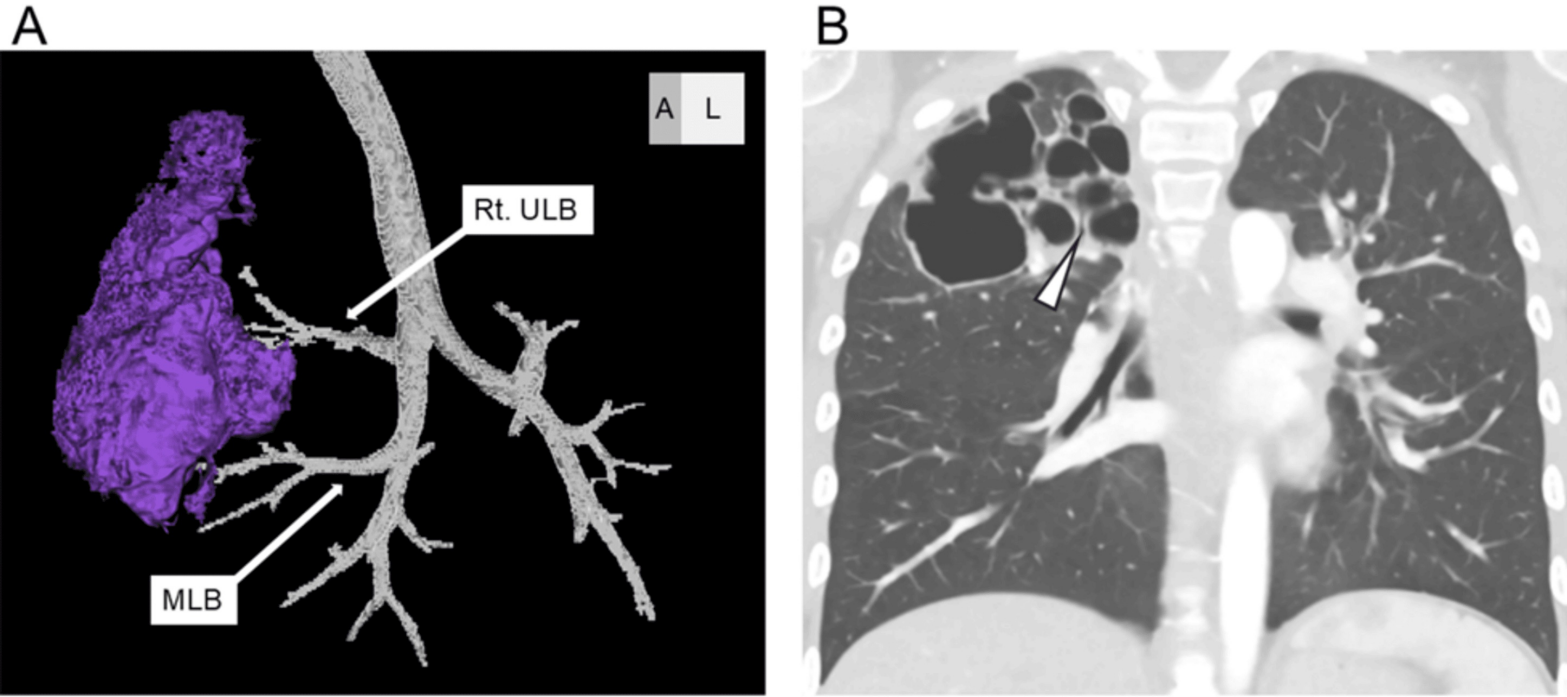
CT images of the cystic lesion (A) Three-dimensional reconstruction showing one of the abscesses extending across the right upper and middle lobes. (B) Coronal HRCT image demonstrating a large cyst with multiple clustered smaller cysts and a connection between the segmental bronchus and the cystic lesion (arrowhead) in the right upper lung field.

On hospital day 22, a contrast-enhanced high-resolution computed tomography (HRCT) scan was performed. The scan showed an absence of normal lobar division between the right upper and middle lobes. Additionally, two distinct multilocular cystic lesions were observed in these lobes. This finding was atypical for a simple bacterial lung abscess and suggested Type 1 CPAM of CCLD as the underlying condition. The cystic lesions measured 64 mm and 46 mm in maximum diameter, respectively, and consisted of approximately ten clustered smaller cysts ranging from 5 to 20 mm in diameter. These cysts communicated with the segmental bronchi (Figure 2).

The lung abscess was considered to have resulted from infection within Type 1 CPAM. After consultation with the pediatric surgery department, a surgical resection of the cystic lesions was planned.

On the same day, the patient developed worsening skin eruptions, raising the possibility of a drug-induced rash. Consequently, the antibiotic regimen was switched to oral clindamycin (CLDM) at a dose of 20 mg/kg/day. The patient was discharged on hospital day 23 and continued regular outpatient follow-up. As some cystic lesions still showed fluid accumulation, oral CLDM therapy was maintained.

**Figure 3:**
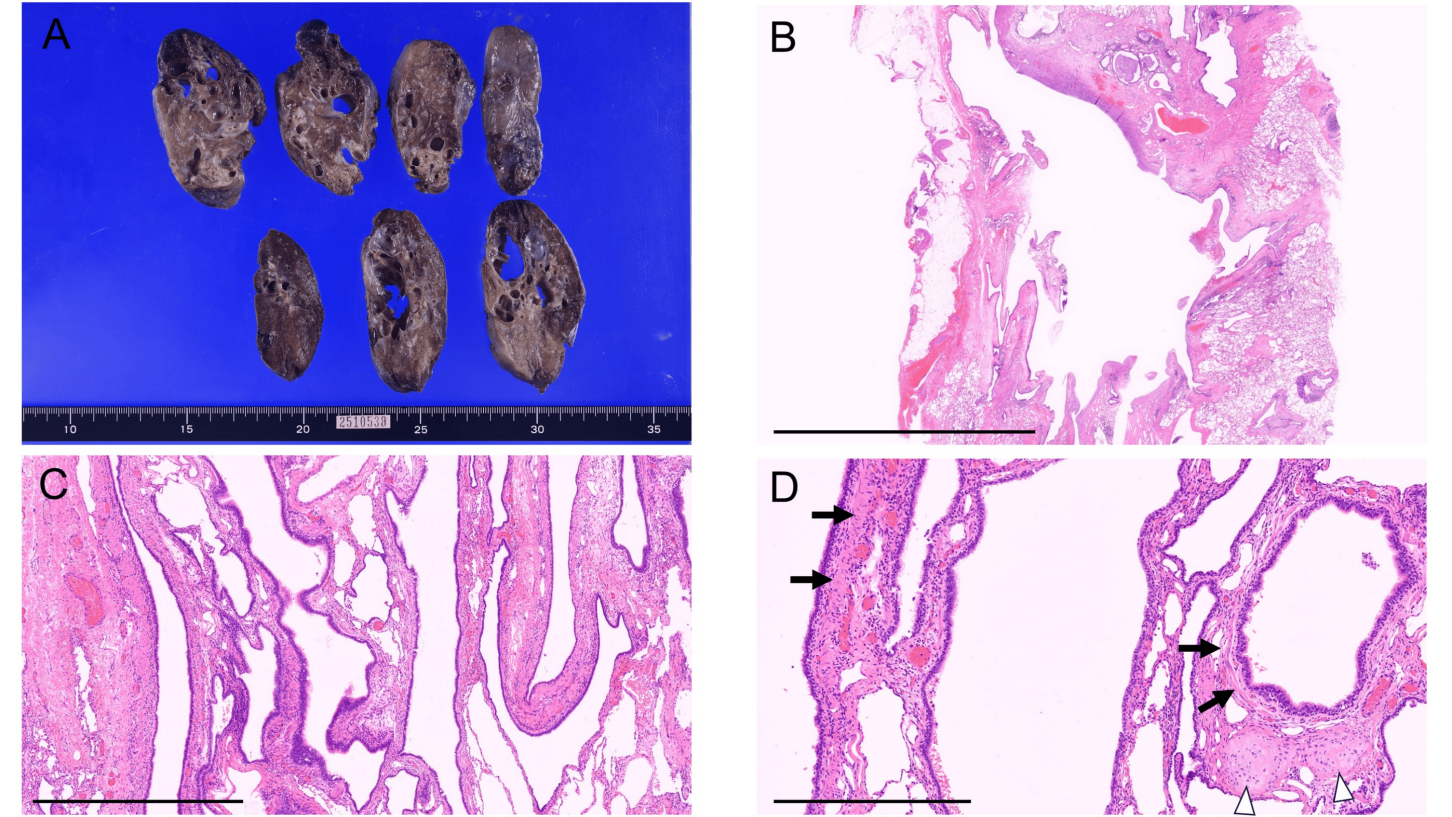
Gross and histopathological findings of the resected lung specimen (A) Gross appearance showing multiple large cysts occupying most of the right upper and middle lobes. (B) Large cystic lesions are visible (hematoxylin and eosin stain, scale bar = 10 mm). (C) Irregularly dilated cysts lined by pseudostratified ciliated columnar epithelium (hematoxylin and eosin stain, scale bar = 1 mm). (D) Smooth muscle tissue (arrow) and small cartilaginous fragments (arrowhead) are observed within the cyst wall (hematoxylin and eosin stain, scale bar = 500 µm).

Several months after admission, the patient underwent right upper lobectomy and wedge resection of the middle lobe. Macroscopically, multiple cysts were observed in the right upper and middle lobes, and histopathological examination confirmed the characteristic features of Type 1 CPAM, including the cyst wall architecture and changes in the surrounding lung tissue (Figure 3).

Consequently, the final diagnosis was confirmed as Type 1 CPAM. Since the surgery, the patient has had a favorable clinical course with no complications to date.

## Discussion

This case involved an eight-year-old girl with no significant history of recurrent pneumonia or respiratory failure, who developed a prolonged fever and respiratory symptoms over approximately two months, eventually leading to the formation of extensive pulmonary abscesses in the right upper and middle lobes. She was hospitalized for further evaluation and required prolonged antibiotic therapy. During the diagnostic workup, no common causative organisms of lung abscess were identified, and immunodeficiency was ruled out. Based on contrast-enhanced CT findings, Type 1 CPAM was suspected. Several months after admission, surgical resection of the cystic lesions was performed, and histopathological examination confirmed the diagnosis of Type 1 CPAM.

Pulmonary abscesses are classified as either primary or secondary based on their pathogenesis. Secondary pulmonary abscesses are known to occur in association with underlying conditions such as aspiration-related disorders (e.g., cerebral palsy, achalasia), immunodeficiency, and congenital cystic lung diseases (CCLD) [4]. In contrast to primary pulmonary abscesses, which are most commonly caused by gram-positive cocci such as Streptococcus pneumoniae and Staphylococcus aureus, secondary abscesses are often associated with different pathogens, including Pseudomonas aeruginosa [4]. Therefore, investigating potential underlying diseases before initiating treatment is essential. When preexisting imaging is available prior to the onset of pneumonia or pulmonary abscess, cystic lung lesions such as CCLD can be more easily suspected. However, in cases like the present one, where there is no history of recurrent pneumonia and no prior imaging, differentiating between a primary pulmonary abscess and one secondary to CCLD can be challenging.

With advancements in fetal ultrasonography, CPAM is increasingly detected around 20 weeks of gestation, often following the identification of cystic thoracic lesions or mediastinal shift [5]. Approximately 80% of congenital cystic adenomatoid malformation (CCAM) cases present with clinical symptoms such as respiratory distress during the neonatal period, leading to early diagnosis [6]. In contrast, cases identified during school age or adulthood are rare. When the cysts are large, they may compress adjacent normal lung tissue, causing respiratory symptoms. Therefore, as in the present case, asymptomatic CPAM with relatively large multilobar cysts detected incidentally during school age is exceedingly uncommon.

In the present case, the pulmonary abscess extended across multiple lobes (Figure 2A). Approximately 10% of congenital thoracic malformations, including CPAM, have been reported to involve more than one pulmonary lobe [7]. In contrast, primary pulmonary abscesses with extensive involvement are rare and typically occur in the context of severe infection or immunodeficiency. Although lesion size alone is not a definitive diagnostic feature, the presence of large lesions should prompt consideration of congenital thoracic anomalies such as CPAM in the differential diagnosis. In this case, the absence of any significant underlying disease initially led to the suspicion of a primary pulmonary abscess. However, since Type 1 CPAM is often associated with milder clinical presentations compared to other subtypes [2], it is possible that the patient had previously experienced unrecognized, self-limiting respiratory infections that did not require hospitalization. This case underscores the importance of a comprehensive diagnostic approach during the acute phase of pulmonary abscess, incorporating a detailed medical history, imaging studies, and immunodeficiency screening. In particular, careful evaluation of patient history and underlying conditions can facilitate more accurate diagnosis and appropriate management in such cases.

Mucinous cell clusters, considered precursor lesions of pulmonary adenocarcinoma, have been identified in approximately 75% of Type 1 CPAM cases [8], and KRAS gene mutations have also been reported in these lesions [9]. These findings indicate a potential for malignant transformation, with an overall malignancy rate of about 4% among congenital pulmonary malformations and a 2% incidence of pleuropulmonary blastoma specifically in CPAM [8]. Furthermore, because the cystic lesions in CPAM often communicate with the bronchial tree, recurrent infections are more likely compared to other congenital cystic lung diseases, such as pulmonary sequestration. Fatal infections, including invasive aspergillosis, have also been documented [10]. Although the optimal timing of surgical intervention for asymptomatic CPAM incidentally detected beyond school age remains uncertain, early surgical resection is generally recommended when CPAM is clinically suspected, given the potential risks of malignant transformation and recurrent pulmonary infections. Further studies are needed to better define long-term outcomes and refine surgical indications in these cases.

## Conclusions

This case describes an unusual presentation of Type 1 CPAM in a school-aged child with no prior history of recurrent pneumonia or respiratory distress. The development of pulmonary abscesses prompted further evaluation, leading to the incidental diagnosis of CPAM, which was subsequently confirmed histopathologically after surgical resection. This report emphasizes the importance of considering congenital cystic lung diseases, including CPAM, in the differential diagnosis of pediatric pulmonary infections, even when presentation occurs at school age. Given the risks of recurrent infection and potential malignant transformation, early recognition and surgical management remain key to achieving favorable outcomes.
